# Functional biology and biotechnology of thermophilic viruses

**DOI:** 10.1042/EBC20220209

**Published:** 2023-08-11

**Authors:** Ryan K. Doss, Marike Palmer, David A. Mead, Brian P. Hedlund

**Affiliations:** 1 School of Life Sciences, University of Nevada, Las Vegas, Las Vegas, Nevada, U.S.A.; 2 Varizymes, Middleton, Wisconsin, U.S.A.; 3 Nevada Institute of Personalized Medicine, Las Vegas, Nevada, U.S.A.

**Keywords:** Coat proteins, DNA replication and recombination, Endolysins, RNA/DNA ligases, Thermophiles, virology

## Abstract

Viruses have developed sophisticated biochemical and genetic mechanisms to manipulate and exploit their hosts. Enzymes derived from viruses have been essential research tools since the first days of molecular biology. However, most viral enzymes that have been commercialized are derived from a small number of cultivated viruses, which is remarkable considering the extraordinary diversity and abundance of viruses revealed by metagenomic analysis. Given the explosion of new enzymatic reagents derived from thermophilic prokaryotes over the past 40 years, those obtained from thermophilic viruses should be equally potent tools. This review discusses the still-limited state of the art regarding the functional biology and biotechnology of thermophilic viruses with a focus on DNA polymerases, ligases, endolysins, and coat proteins. Functional analysis of DNA polymerases and primase-polymerases from phages infecting *Thermus*, *Aquificaceae*, and *Nitratiruptor* has revealed new clades of enzymes with strong proofreading and reverse transcriptase capabilities. Thermophilic RNA ligase 1 homologs have been characterized from *Rhodothermus* and *Thermus* phages, with both commercialized for circularization of single-stranded templates. Endolysins from phages infecting *Thermus*, *Meiothermus*, and *Geobacillus* have shown high stability and unusually broad lytic activity against Gram-negative and Gram-positive bacteria, making them targets for commercialization as antimicrobials. Coat proteins from thermophilic viruses infecting *Sulfolobales* and *Thermus* strains have been characterized, with diverse potential applications as molecular shuttles. To gauge the scale of untapped resources for these proteins, we also document over 20,000 genes encoded by uncultivated viral genomes from high-temperature environments that encode DNA polymerase, ligase, endolysin, or coat protein domains.

## Introduction

Life in high-temperature environments poses challenges that are met by adaptations that increase the stability of all macromolecules, including proteins. The same properties that make thermophilic proteins vital to their thermophilic hosts—intrinsic stability and activity at high temperatures—also offer important advantages over their mesophilic counterparts for industrial and molecular biology applications. As a classic example, Taq polymerase, isolated from *Thermus aquaticus*, was employed in the 1980s to substitute for *Escherichia coli* DNA polymerase for the polymerase chain reaction (PCR) [1]; the stability of Taq polymerase under conditions required to thermally denature DNA improved the practicality and costs of PCR, and was critical for its rapid expansion as a cornerstone of modern molecular biology, disease diagnostics, forensics, and genetic genealogy, among other technologies [2]. High temperatures also decrease nucleic acid secondary structures, off-target base-pairing, and nonspecific protein–protein and ligand–protein interactions, thereby improving the efficiency and fidelity of a wide variety of biochemical interactions. Thermophily also increases compatibility with a variety of industrial and molecular biology applications, including better performance in viscous solutions, which become more fluid at higher temperature, and increasing volatility of biofuels [3,4]. In addition, thermophilic enzymes are typically more stable than mesophilic enzymes [5], which can increase shelf-life, enhance stability under a variety of extreme conditions, and simplify purification schemes; for example, heat purification of thermostable enzymes is a simple method for isolating recombinant thermophilic proteins from crude lysates of mesophilic expression systems [6–8].

Even though biotechnology has long relied on enzymes from thermophilic prokaryotes, those from thermophilic viruses that infect them remain remarkably underexplored. All viral genomes encode key enzymes that are necessary for the biology of the virus, including those involved in diversion of host resources for viral genome replication, transcription and translation, evasion of host immunity, packaging, and egress from the host cell [9,10]. Genes encoding these functions occur at much higher frequencies in viral DNA than in host DNA and high recombination rates within viruses promote biochemical innovations. In all, these biochemical innovations provide excellent targets for biotechnological commercialization.

Over the last two decades, rapid progress has been made on the cultivation and molecular biology of thermophilic and hyperthermophilic host–virus pairs, especially among novel archaeal viruses infecting the thermoacidophilic order *Sulfolobales* and to a lesser extent thermophilic *Thermoproteales*, both belonging to the phylum *Thermoproteota* (synonym *Crenarchaeota*) [11,12]. These archaeal viruses belong to the International Committee on Taxonomy of Viruses (ICTV) families of *Lipothrixviridae*, *Rudiviridae*, *Tristomaviridae*, *Turriviridae*, *Ampullaviridae, Bicaudaviridae*, *Spiraviridae, Fuselloviridae*, *Guttaviridae*, *Clavaviridae*, and *Globuloviridae* [11,13]. Parallel research on thermophilic bacteriophages has largely focused on those infecting the genera *Thermus*, *Meiothermus*, *Geobacillus*, and *Rhodothermus* [14]. Most of these bacteriophages belong to the class *Caudoviricetes* (recently reclassified based on genomic information and not morphology [15]), while others represent novel families not yet placed in higher taxonomic ranks or are largely unclassified (e.g., unclassified myo- and siphoviruses ϕYS40, G20c, and RM378). These viruses replicate at high temperatures and are often stable at temperatures exceeding the optimal growth temperatures of their host thermophiles or hyperthermophiles [16–18]. However, viral genomes are also universally enriched in poorly annotated genes [19], with tens of thousands of poorly annotated small-gene families being discovered recently [20]. Together, these genes represent a vast, underexplored resource with potential to contribute innumerable advances in biotechnology and biomedicine.

**Figure 1:**
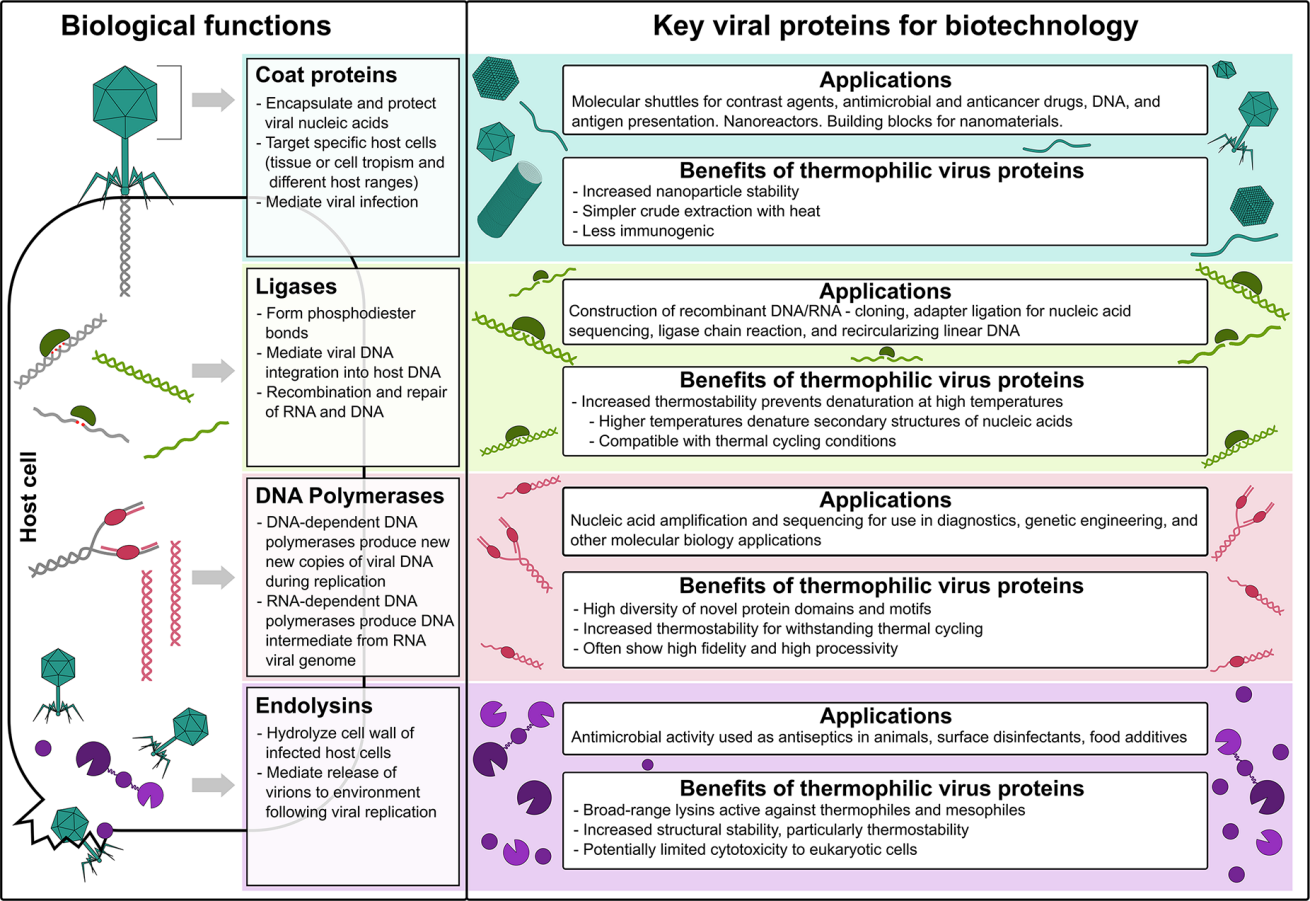
Graphical summary of proteins discussed. Biological functions and key biotechnological applications of thermophilic viral coat proteins, ligases, DNA polymerases, and endolysins are highlighted.

This paper highlights a relatively small number of functionally characterized proteins from thermophilic bacteriophage and archaeal viruses and their potential roles in biotechnology, focusing on proteins with potential applications in DNA synthesis, nucleotide modifications and repair, cell lysis, and nano-trafficking (Figure 1 and Table 1). We also provide an up-to-date accounting of putative proteins of biotechnological interest in uncultivated viral genomes (UViGs) from thermal environments and discuss key opportunities to explore these proteins for biotechnology purposes (Table 2, Supplementary Material).

**Table 1:** Functionally characterized proteins from thermophilic viruses and their potential biotechnology applications

Proteins	Characterized thermophilic viral protein	Source virus (Synonyms)	Functional or structural characterization and biotechnology applications to date	Accession number	References
DNA Polymerases	vB_Tt72 PolA	Unclassified myovirus vB_Tt72 (*Thermus* phage vB_Tt72)	**Functional:** Verified 3′-5′ exonuclease activity, nucleotidyltransferase domain, and performed optimally at 55°C and pH 8.5.	ON714139.1	[27]
	PolI_G20c	Unclassified *Oshimavirus* G20c (*Thermus* phage G20c)	**Functional:** Structurally and functionally characterized confirming 3′-5′ exonuclease activity and DNA polymerase activity, with optimal polymerase activity at 70°C at pH 9.1.	KX987127.1	[30]
	3173 Pol	Metagenomic fragment from ‘Pyrovirus’ from Octopus Spring, Yellowstone	**Functional:** Verified 3′-5′ exonuclease activity and DNA-dependent DNA polymerase activity and RT activity with optimum of 77°C and half-life of ∼11 min. at 94°C. **Biotech:** One-enzyme RT-PCR.	ADL99605.1	[26]
	OCT 1608-14 Pol	PCR amplified from metagenomic DNA from Octopus Spring, Yellowstone; full-length variant of 3173 Pol from ‘Pyrovirus’	**Functional:** DNA polymerase activity confirmed under conditions similar to those of 3173 Pol. (Contains N-terminal DUF927 domain in addition to 3′-5′ exonuclease/DNA polymerase A domain of 3173 Pol.)	KC440900	[38]
	LavaLAMP/ PyroPhage 3173 PolA	Engineered fusion of ‘Pyrovirus’ 3173 Pol and Sso7d polymerase	**Functional:** Verified 3′-5′ exonuclease activity and DNA-dependent DNA polymerase activity and RT activity with optimum of 85°C. **Biotech:** RT-PCR, RT-LAMP, cDNA cloning for RNA Seq. Commercialized by Lucigen Co. as LavaLAMP/PyroPhage 3173 PolA.	AFN99414.1	[25,26,37,40]
	Magma DNA polymerase	Chimera of shuffled ‘Pyrovirus’ Pols with Taq polymerase 5′-3′ exonuclease	**Functional:** Verified higher fidelity over other 3173 Pol variants/relatives (1 error per 10^6^ nucleotides), increased primed-template binding affinity. **Biotech:** Optimized for single-enzyme RT-PCR.	US 2021/0171580 A1^a^	[40]
	NrS-1 primase-polymerase	Unclassified siphovirus NrS-1 (*Nitratiruptor* phage NrS-1	**Functional:** Verified polymerase, primase, and helicase activity at 50°C. Lacks finger and thumb subdomains. **Biotech:** Primer-free DNA synthesis for whole genome amplification	BAN05337.1	[42–44]
Ligases	RM378 RNA ligase	Unclassified myovirus RM378 (*Rhodothermus* phage RM378)	**Functional:** Experimentally determined high specificity (10x higher than T4 RNA ligase), and RNA/ ssDNA ligation optimally at 64°C and pH 6-7. **Biotech:** Patented and commercialized as a competitor to RLM-RACE for cDNA ligation.	NC_004735.1	[61]
	TS2126 RNA ligase	Unclassified virus Ph2119 (*Thermus* phage Ph2119)	**Functional:** Experimentally determined high specificity (30x higher than T4 RNA ligase), and RNA/ssDNA ligation optimally at 65°C and pH 7.5. **Biotech:** Commercialized as CircLigase™ for cDNA ligation and circularization of linear nucleic acids by Epicentre.	C0HM52.1	[62,63]
Endolysins	Ts2631 endolysin	Unclassified virus vB_Tsc2631 (*Thermus* phage vB_Tsc2631)	**Functional:** *In vitro* antibacterial activity against Gram-positive and Gram-negative bacteria performing optimally at 40–105°C and pH 7.0–11.0.	KJ561354	[69,71]
	Ph2119 endolysin	Unclassified virus Ph2119 (*Thermus* Phage Ph2119)	**Functional:** *In vitro* antibacterial activity against Gram-negative bacteria performing optimally from 50°C to 105°C and pH 7.5–8.0.	KF408298.1	[70]
	TSPphg endolysin	Unclassified *Oshimavirus* TSP4 (*Thermus* phage TSP4)	**Functional:** *In vivo* clinical testing in mice infected with multidrug-resistant *Staphylococcus aureus* reduced infection performing optimally at 40–70°C and pH 7.0–10.0.	QAY18185	[73,75]
	MMPphg endolysin	Unclassified myovirus MMP17 (*Meiothermus* phage MMP17)	**Functional:** *In vitro* antibacterial activity against Gram-positive and Gram-negative bacteria. This enzyme performed optimally from 35°C to 65°C.	QAY18044	[74,75]
	MLTphg	Chimera of TSPphg and MMPphg	**Functional:** *In vitro* antibacterial activity against Gram-positive and Gram-negative bacteria greater than TSPphg and MMPphg alone optimally from 35 to 40°C.	–	[75]
	GVE2 endolysin	Unclassified siphovirus GVE2 (*Geobacillus* phage GVE2)	**Functional:** Verified lysis against *Geobacillus* sp. E263 through interaction with a phage holin and host ABC transporter. This enzyme was active at 60°C.	YP_001285830.1	[77]
	GVE2_CAT_-fusions	*Geobacillus* sp. E263 phage GVE2 catalytic domain fused with several *Clostridium perfringens* phage cell-wall binding domains	**Functional:** *In vitro* analysis revealed antibacterial activity against *Clostridium perfringens*, performing above 50% activity from 4 to 60°C. **Biotech:** Patented for commercialization	–	[78]
	TP-84 endolysin	*Saundersvirus* Tp84 (*Geobacillus* Phage TP-84)	**Functional:** *In* vitro analysis revealed biofilm reduction against Gram-positive and Gram-negative bacteria with full activity at 30–75°C.	YP_009600073.1	[76]
Coat Proteins	SIRV2 capsid protein	*Icerudivirus* SIRV2 (*Sulfolobus islandicus* rod-shaped virus 2 SIRV2)	**Functional:** Experimentally determined stability range of -80°C to 80°C at pH 6. Cryo-EM structure. **Biotech related:** SIRV2 stability was monitored in different solvents, and attachment sites and ligands identified.	NP_666560.1	[95,96]
	SMV1 coat protein	Unclassified *Bicaudaviridae* SMV1 (*Sulfolobus* monocaudavirus 1 SMV1)	**Functional:** *In vivo* stability without inflammatory response; passed through simulated gastric fluid, GI tracts of mice, and human intestinal organoids; vector stability and immunogenicity assessed.	YP_009008070.1	[94]

a Sequence available in this US patent.

**Table 2:** Summary of protein families (Pfams) counts associated with the genes of interest found in UViGs from thermal environments in the IMG/VR v4 database [99] (January 25, 2023)^a^

Protein Category	Pfams queried	Marine thermal systems		Terrestrial thermal systems		Total
		Hydrothermal vents	Marine volcanic	Thermal Springs-Warm/Hot/ (42–90°C)	Other terrestrial geothermal^b^	Sum per protein category
**DNA Polymerases**	00078, 00136, 00476, 03175, 08996, 10391, 14791, 14792, 20286	2,889	567	1,557	33	5,046
**Ligases**	01068, 01653, 03119, 03120, 04675, 04679, 09414, 09511, 11311, 13298, 14743, 18043	2,849	185	1,058	19	4,111
**Endolysins**	00959, 01183, 01476, 01510, 11125, 11860, 18341	2,024	381	2,102	6	4,513
**Coat/Capsid Proteins**	01819, 02305, 03864, 05065, 05356, 05357, 06152, 06673, 07068, 09018, 09063, 09300, 10665, 11651, 12691, 16710, 16855, 16903, 18628, 19199, 19307, 19821, 20036	5,264	608	4,272	49	10,193
**Sum per environment**	-	13,026	1,741	8,989	107	23,863
**Total UViGs per environment**	-	110,140	19,154	55,911	2,547	187,752

a See **Supplementary Material** for detailed information.

b Sum of the following IMG/VR categories: Thermal Springs-Runoff channels, Sediment-Thermal Springs, and Volcanic Fumaroles.

## DNA polymerases

Thermophilic DNA polymerases have been a focal point of development and commercialization for biotechnology companies due to their versatility, with uses ranging from molecular diagnostics to next-generation DNA sequencing. In 1988, Taq polymerase, a family-A DNA polymerase (PolA) from *T. aquaticus*, was optimized for PCR due to its thermophily, with the important caveat that it has a high error rate at one mutation per 20,000 base pairs [21,22]. Bacteriophage ϕ29 DNA polymerase is a mesophilic enzyme with lower error rates [23] that allows for isothermal amplification with proofreading, high processivity, and strand-displacement capabilities, but it only functions at low temperatures. Attempts to identify thermophilic viral DNA polymerases similar to Taq polymerase, but that can proofread, or to identify a suitable thermophilic alternative to ϕ29 polymerase would improve the capabilities of existing applications. Thus far, some thermophilic viral DNA polymerases have been identified that contain high-fidelity proofreading domains, strand-displacement, or reverse-transcriptase activity [22,24–26].

Several thermophilic viral alternatives to Taq polymerase have been expressed, purified, and characterized (Table 1), including two originating from bacteriophage infecting the genus *Thermus*. Many cultivated *Thermus* phage encode annotated family-A DNA polymerase (*polA*) genes, including vB_Tt72 [27], ϕYS40 [28], ϕTMA [29], G20c [30], P7426 [31], P2345 [31], ϕFA [32], TSP4 [33], and Tth15-6 [34]. Of these, the unclassified myoviruses *Thermus* phage vB_Tt72, phage ϕYS40, and phage ϕTMA are closely related and likely represent a novel genus in the *Caudoviricetes* [27]. They have similar *polA* genes (>90% amino acid identity) with low sequence similarity to other polymerases [27]. Biochemical characterization of the vB_Tt72 DNA polymerase revealed proofreading activity even at low dNTP concentrations (0.4 mM), properties that Taq polymerase lacks completely [22]. However, the purified vB_Tt72 polymerase lost function rapidly above 60°C, and was not thermostable enough for PCR [27], limiting potential biotechnology applications.

*Thermus* phages G20c, P7426, P2345, ϕFA, and TSP4 were isolated from terrestrial springs on several continents. All belong to the genus *Oshimavirus* [35] and they have similar *polA* genes. The PolA from G20c, PolI_G20c, has been both structurally and functionally characterized [30]. It was shown to be structurally similar to Taq polymerase, consisting of 3′-5′ exonuclease, helicase, and PolA domains, including a novel motif named SβαR near the exonuclease domain believed to play a role in substrate binding [30]. Both DNA polymerase and 3′-5′ exonuclease activities were experimentally verified, with maximal DNA polymerase activity at 70°C, which is too low for thermocycling-based biotechnology applications.

In addition to these cultivated viruses, a prophage-encoded DNA polymerase within the chromosome of *Thermus antranikianii* was characterized and shown to have strong strand-displacement activity, similar to ϕ29, but many of the amplification products were highly branched, non-specific DNA molecules. Thus, this polymerase is not suitable as a thermophilic alternative to ϕ29 [24], but this prophage polymerase shows that thermophilic strand-displacement is possible. A thermophilic polymerase with properties similar to ϕ29—high fidelity, high processivity, and strong strand-displacement activity—would enable high-fidelity, long-range PCR and would be of considerable biotechnology interest.

Aside from work on cultivated viruses, bioinformatic [36,37] and functional screens [25,26] for DNA polymerases in viral metagenomic DNA from diverse terrestrial hot springs revealed a group of 3′-5′ proofreading exonuclease and DNA polymerase (3′ exo/pol)-encoding genes within metagenomic fragments and UViGs assigned to the putative genus ‘Pyrovirus’, which is predicted to infect genera within the *Aquificaceae*. Comparative phylogenetics showed these unusual *polA*s spread by horizontal gene transfer among thermophilic viruses, their *Aquificota* hosts, other diverse bacteria (although only temporarily retained), and the proto-apicoplast that became a symbiotic partner of an ancestor to the eukaryotic phylum *Apicomplexa* [38]; yet, only the viral enzymes encode N-terminal helicase domains (DUF927). The interdomain lateral gene transfers of these large and unique *polA*s suggest they may be associated with dispersal of diversity-generating mechanisms between geothermal and moderate-temperature biomes [38]. An engineered fusion between a ‘Pyrovirus’ PolA enzyme with the *Sulfolobus solfataricus* Sso7d DNA binding protein [39], called PyroPhage 3173 PolA, was shown to have three novel characteristics, namely (i) reverse transcriptase (RT) activity, (ii) DNA polymerase strand-displacement activity, and (iii) thermostability, which enabled RT-PCR and reverse transcription loop-mediated isothermal amplification (RT-LAMP) [25,26]. PyroPhage 3173 PolA had an error rate approximately 10 times lower than Taq polymerase, and the enzyme was commercialized by Lucigen Corporation (acquired by LCG, Middleton, Wisconsin). Amino acid swapping with *polA* genes from related UViGs, and domain swapping with the Taq polymerase 5′- 3′ exonuclease resulted in a recombinant enzyme, called Magma DNA polymerase, that was more thermophilic, more accurate (as low as 1 error in 10^6^ nucleotides), and performed better in reverse-transcriptase PCR applications [40].

Viral DNA-directed primase-polymerase-like proteins are predicted to have additional roles in DNA and/or RNA priming, as well as damage-tolerant DNA polymerase activity [41], and at least one such unusual thermophilic enzyme from unclassified deep-sea vent phage NrS-1, infecting *Nitratiruptor* sp. SB155-2 [42], was shown to be functional [43,44]. This enzyme has features found in DNA polymerases (DNA-dependent polymerization), primases (primer-free DNA strand synthesis initiation), helicases (strand displacement), and RNA polymerases (RNA-dependent polymerization), and could be useful in several potential applications, such as primer-free, isothermal whole-genome amplification.

## Ligases

DNA and RNA ligases catalyze the formation of phosphodiester bonds between 5′-phosphate and 3′-hydroxyl groups, with activity on DNA or RNA, respectively [45]. These enzymes serve critical functions *in vivo*, including DNA replication and recombination, somatic generation of immune diversity, nucleic acid editing, and DNA/RNA repair (Table 1). Biotechnology applications of ligases include decades-old technologies such as construction of recombinant plasmids or viruses, but also emerging technologies such as library preparation for DNA and microRNA sequencing [46], single nucleotide polymorphism diagnostics using the ligase chain reaction [47], and synthetic gene construction via Gibson assembly, a cornerstone technology of modern synthetic biology [48].

Most biotechnology applications use ligases from bacteriophage T4 [46,47], but T4 DNA and RNA ligases are incompatible with denaturation conditions necessary for ligation chain reaction (90°C) [49–51], are unstable at temperatures used for Gibson assembly (typically 50°C) [48], have limited efficiency due to competition with secondary structures [52], and have low fidelity due to off-target base pairing [52]. More than 25 ligases from archaea have been functionally characterized, with possible biotechnology applications including Gibson assembly [53–55], ligase chain reaction [54,56], 5′-adenylation [57,58], and RNA sequencing [59]. These enzymes have recently been reviewed [60].

Comparatively less work has been done to characterize ligases from thermophilic viruses, and all published work to date has focused on moderately thermophilic ATP-dependent RNA ligase 1 enzymes. In 2003, a thermophilic homolog of T4 RNA ligase 1 from *Rhodothermus* phage RM378, an unclassified myovirus, was shown to have optimal activity at 64°C [19,61]. This enzyme could substitute for T4 RNA ligase 1 in RNA ligase-mediated rapid amplification of cDNA ends (RLM-RACE) at 60°C and was patented and commercialized for that purpose. In 2005, another thermostable RNA ligase 1 from the unclassified virus *Thermus* phage Ph2119, TS2126 RNA ligase, was also characterized [19,62]. The TS2126 RNA ligase had ∼30 times higher specific activity compared with T4 RNA ligase in phosphatase protection assays with a temperature optimum of 70-75°C and it was also more effective with ssDNA ligation. This enzyme complexes with adenylated donors rapidly, with a slower ligation activity, which strongly favors intramolecular ligations. This property has been exploited for 5′ preadenylylation of DNA oligonucleotide adapters during cDNA library preparation [63] and for circularization of single-stranded DNA or RNA templates for rolling-circle replication or rolling-circle transcription experiments. Kits for the latter produce virtually no linear or circular concatemers and have been trademarked as CircLigase™ ssDNA Ligase and CircLigase™ II ssDNA Ligase by Epicentre (acquired by Illumina, Madison, WI, U.S.A.).

## Endolysins

All viruses require mechanisms to escape infected host cells following replication and assembly, and enzyme systems for this purpose are as diverse as the cell envelopes of their host prokaryotes [64–66]. Endolysins of mesophilic bacteriophages are hard to purify, have limited stability and activity under industrial conditions, and are typically highly specific, with many showing activity only against one host species or just a group of strains [67,68]. These shortcomings limit their use as antimicrobials, yet some evidence suggests these limitations may be overcome by their thermophilic counterparts. Currently, several native endolysins from thermophilic viruses and thermophilic recombinant endolysins have been investigated for broad-range applications (Table 1).

Two endolysins from unclassified cultivated phages infecting *T. scotoductus* strain MAT2119, phage vB_Tsc2631 and phage Ph2119, were shown to be homologs of T3 and T7. These endolysins lysed *Thermus* strains, *Deinococcus radiodurans*, and also Gram-negative mesophiles such as *E. coli*, *Salmonella enterica*, *Serratia marcescens*, and *Pseudomonas fluorescens* [69,70]. The Ph2119 endolysin retained 87% activity at 95°C, and the endolysin from vB_Tsc2631 retained 65% activity at 95°C. The endolysin from vB_Tsc2631 has seven charged arginine amino acids near the N-terminus, making the endolysin act like polycationic antibacterial peptides that form pores in cell membranes, allowing for the catalytic center of the endolysin to interact with the peptidoglycan underneath [71]. Arginine is also more thermostable than other positively charged amino acids because its functional group mimics guanidinium [71,72]. The active site of the vB_Tsc2631 is also believed to bind Zn^2+^ ions for catalytic functions and structural stability. These properties are natural advantages necessary for thermophily that overcome many of the obstacles that limit the utility of mesophilic endolysins as antimicrobials.

The endolysin of *Oshimavirus* TSP4 (also known as *Thermus* phage TSP4), TSPphg, was expressed in *E. coli*, purified, and shown to reduce *Staphylococcus aureus* infections in mice, offering promise for clinical treatments for bacterial infections [73]. Further *in vitro* testing showed antimicrobial activity against Gram-negative *S. enterica, Klebsiella pneumoniae, E. coli*, and Gram-positive *Bacillus subtilis*. The broad antimicrobial activity of TSPphg may be due to strong interactions with peptidoglycan, owing to the six positively charged amino acids near the N-terminus, similar to the endolysin of vB_Tsc2631 [70,73].

Another broad range endolysin, MMPphg, was found in *Meiothermus* phage MMP17, an unclassified myovirus. Purified MMPphg has optimal activity at 65-70°C and lyses both Gram-positive and Gram-negative bacteria, including *E. coli, S. aureus, S. enterica*, and *Shigella dysenteriae*, and eight different antibiotic-resistant strains of *K. pneumoniae* [74]. The C-terminus of the lysin also contains six positively charged amino acids. The MMP17 endolysin was later artificially fused with TSPphg and the recombinant enzyme, MLTphg, showed higher antimicrobial activity *in vitro* than either individual endolysin [75].

The other two functionally characterized endolysins are from viruses infecting Gram-positive thermophiles. GVE2, an unclassified siphovirus that infects *Geobacillus* sp. E263, encodes an endolysin believed to interact with a host eukaryotic-type ABC transporter to lyse host cells at temperatures from 55 to 90°C [76,77]. Because this endolysin is the first known to interact with ABC transporter proteins, it was further investigated as a potential antimicrobial [78]. The catalytic domain of the GVE2 endolysin was fused to peptidoglycan-binding domains from endolysins of several different *Clostridium perfringens* viruses. The result was chimeric endolysins that could operate up to 70°C, making these enzymes a potential antibiotic treatment for animals that can be added to their heat-sterilized feed [78].

Recently, an endolysin from *Saundersvirus* Tp84 (also known as *Geobacillus* virus TP-84) was investigated as a potential disinfectant for surfaces at high temperatures [79]. This endolysin had activity throughout the temperature range of 30-70°C and inhibited biofilm formation by *Pseudomonas aeruginosa*, *Streptococcus pyogenes*, and *S. aureus*. Extensive human safety testing is recommended for all endolysins to ensure they are safe for consumption if used as additives, and further testing on the long-term stability of these endolysins would be required to evaluate their potential use [78,79].

Phage depolymerases, including both hydrolases and lyases, have been investigated for their ability to degrade polysaccharides or lipids depending on the host's envelope [80–82]. Recent research has suggested that a cocktail of endolysins and envelope depolymerases would produce a greater antimicrobial effect on biofilm-forming bacteria such as *P. aeruginosa* [81,83,84]; however, investigation of thermophilic envelope depolymerases remains sparse. Although several genes from thermophilic phages are annotated as encoding some form of envelope depolymerase [84], evidence for expression of these enzymes are limited to the formation of halos around clear plaques in eight thermophilic *Geobacillus* phages, including TP-84 [79,84]. Due to the relative lack of functional studies performed on the efficacy of thermophilic depolymerases and endolysin cocktails, this presents a notable target for research and development of possible biotechnological applications.

## Coat proteins

Viruses consist of nucleic acids encapsulated by protein coats (capsids) that comprise numerous copies of one or more coat protein subunits. As part of the virion, capsids protect nucleic acids [85,86], serve as vehicles for transport that can target specific cells [85], and mediate introduction of nucleic acids into host cells during infection [85,86]. As these particles typically have natural tropism toward certain cell types, and are often resistant to immune defense systems, coat proteins are excellent candidates as nano-traffickers in biomedicine. These coat proteins self-assemble [60,85,87] and inclusion of certain protein domains within them can result in highly specific targeted delivery of compounds [85,88,89].

A variety of molecules can be encapsulated by capsids, resulting in virus or virus-like nanoparticles [19,59,85,90]. These nanoparticles can be used in biomedicine for delivery of contrast agents for medical imaging [85,88,89,91], anticancer or antimicrobial drugs [85,88,89,91], antigen-presenting platforms for vaccines [85,87,91], or engineered shuttles for genetic material in gene therapy [85,91,92]. Additionally, these capsids can also serve as nanoreactors [85,89,90] and can be used in non-medical applications like transport of inorganic compounds and production of nanomaterials [85,93]. However, limitations of capsids from mesophilic viruses infecting animals, plants, or mesophilic microorganisms include their limited chemical and physical stability [85,94], challenges purifying nucleic acid-free capsids from infected host cells or expression hosts [90], and residual immunogenicity [85,89,94], which may result in clearance of nanoparticles from the system before achieving the desired effect [85,94]. Several of these shortcomings can be alleviated by capsids derived from thermophilic viruses (Table 1).

The first thermophilic viral capsids tested for chemical and physical stability in different solvents, and for availability of ligand attachment sites, were those from *Icerudivirus* SIRV2 (also known as *Sulfolobus islandicus* rod-shaped virus 2) [95]. To assess the stability of SIRV2 particles, the structural integrity and infectivity of virions was assessed following incubations in DMSO and ethanol. SIRV2 particles remained intact and infective for 6 days in 20% ethanol, 20% DMSO, or 50% DMSO, respectively, and remained intact in up to 50% ethanol [95]. With its high stability in DMSO, a solvent commonly used for bioconjugation applications, the availability of ligand attachment sites was evaluated through biotinylation of the SIRV2 particles using different compounds to identify reactive carboxylates, carbohydrates, and amines [95]. With amine reactivity found only in the minor coat protein subunits, located at the ends of the rod-shaped virions, and reactive carboxylates and carbohydrates found in both minor and major coat protein subunits, a broad variety of functional groups can be conjugated to these viral nanoparticles, including spatially specific selective bioconjugation to only the minor coat protein subunits [95,96].

More recently, an unclassified virus in the *Bicaudaviridae*, *Sulfolobus* monocaudavirus 1 (SMV1), was tested extensively for potential biotechnology applications, especially as a potential nano-trafficker for biomedical use. SMV1 particles were treated with ethanol, DMSO, simulated gastric fluid, and simulated intestinal fluid solutions to assess stability through *S. islandicus* plaque assays, and particles remained infective for up to 6 days [94]. Subsequently, SMV1 particles were passed through the gastrointestinal tracts of mice or incubated with human intestinal organoids, where limited immune responses were elicited, and no SMV1 particles were detected in off-target organs or tissues [94]. Overall, SMV1 particles fared better than *Inovirus* M13KE, an *E. coli* phage used for comparison, in both the mice and organoids [94], showing great promise as both molecular delivery systems and antigen-presentation platforms.

## Expansion of bioprospecting through viral metagenomes

Most research to date has focused on only viruses infecting cultivated thermophilic archaea and bacteria [14,19], thus limiting the overall breadth of our understanding of the thermophilic virome. Given the limited diversity of cultivated thermophilic prokaryotes [97,98] and their viruses [14], and the extremely limited number of biochemically characterized proteins from thermophilic viruses, we propose that UViGs represent a vast resource for the biotechnology sector. To begin to evaluate the potential resource, we searched for pfams that are diagnostic of the four protein groups discussed here—DNA polymerases, ligases, endolysins, and coat proteins—in the Integrated Microbial Genomes/Virus (IMG/VR) v4 database, which contains over 5.5 million high-confidence viral genome contigs from a wide range of biomes [99], focusing on marine and terrestrial geothermal systems (Table 2; Supplementary Material). This search revealed >20,000 potential matches to these four protein groups from >185,000 UViGs, with the largest amount coming from marine hydrothermal systems, followed by high-temperature terrestrial geothermal systems, and the largest protein category being coat proteins, followed by polymerases, endolysins, and ligases. For example, we identified over 5,000 putative polymerases; the vast diversity of polymerase architectures driven by adaptation to thermal environments [88] are ripe for biotechnology exploration.

Despite the vast resources available in UViGs, there are currently some limitations. First, given the genetic and biochemical diversity encoded by UViGs, a vast diversity of biotechnologically useful functions resides in poorly annotated genes that are difficult to bioprospect based on sequence similarity. This hidden resource could be systematically explored using artificial intelligence platforms, including those examining protein folds, which are more highly conserved than primary sequence information [100]. Another limitation is the systematic focus on dsDNA viruses due to predominant library preparation methods used for viral metagenomics, which unfortunately exclude six of the seven groups of the Baltimore classification system [101,102]. This heavy focus on dsDNA viruses ignores many novel architectures of undiscovered viruses and certainly biases our understanding of the thermophilic virome, although known recombination between natural virus populations with different types of genomes may relieve this limitation to a degree [103,104]. Groups such as the RNA Virus Discovery Consortium have deposited many more RNA viral metagenomes into IMG/VR [99] through RNA extraction and reverse transcription prior to or during library preparation, with advancements to date in marine [105,106], sediment [107], and terrestrial ecosystems [108,109], including thermal springs and others [107,110]. Bioinformatic pipelines like VirSorter are also increasing accuracy and providing support for RNA viruses [111]. A separate problem is the identification and classification of metagenomic contigs as UViGs in the first place, which can lead to both false negatives and false positives, although community standards have been developed to improve communication of UViG quality, with UViGs categorized as high quality (>90% completeness), medium quality (50–90% completeness), low quality (<50% completeness), and unsure quality (>120% or no completeness estimate) [99,112]. Despite these challenges, we contend that UViGs provide an immense and poorly explored resource for bioprospecting the global thermophile virome.

In recognition of this resource, projects focused on exploring the sequence coverage of the virosphere are seeing increasing support. This is evident in community-driven sequencing efforts supported by the Joint Genome Institute (e.g., OSTI 1488193, Award 503441), implementation of analysis tools for viruses in collaborative cyberinfrastructure, like CyVerse [113], and the RNA Virus Discovery Consortium [110]. Additionally, in 2016 to 2020, the European Union funded the Virus-X project—Viral Metagenomics for Innovation Value—at €8 million. These projects have expanded the sequence coverage of the global virosphere, expressed and characterized novel proteins [114–116], analyzing crystal structures of expressed genes to aid in functional identification [117], and improved methods to identify and interpret UViGs [111,113], including algorithms to identify host-virus pairs [118] and to improve annotation of uncharacterized viral genes through protein clustering [119,120]. These advancements show not only that thermophilic viral enzymes are an expanding topic of importance for biotechnology, but also that infrastructure and data mining tools are improving to better support the ever-expanding UViG dataset.

## Summary

- Viral proteins, particularly polymerases, ligases, endolysins and coat proteins, provide a bountiful but underutilized toolbox for the biotechnology industry to explore.
- Applications involving thermophilic viral proteins provide several benefits that overcome some of the shortcomings of their mesophilic counterparts.
- Bioprospecting of genomes from uncultivated viruses provides a vast and underexplored resource that overcomes the primary impediment of cultivability.

## Abbreviations

ICTV: International Committee on Taxonomy of Viruses
IMG/VR: Integrated Microbial Genomes/Virus
PCR: polymerase chain reaction
Pfams: protein families
RT-LAMP: reverse transcription loop-mediated isothermal amplification
RLM-RACE: RNA ligase-mediated-rapid amplification of cDNA ends
SIRV2: *Sulfolobus islandicus* rod-shaped virus 2
SMV1: *Sulfolobus* monocaudavirus 1
UViG: uncultivated viral genomes
